# Arbuscular mycorrhizal fungi enhance photosynthesis, water use efficiency, and growth of frankincense seedlings under pulsed water availability conditions

**DOI:** 10.1007/s00442-012-2258-3

**Published:** 2012-01-28

**Authors:** Emiru Birhane, Frank J. Sterck, Masresha Fetene, Frans Bongers, Thomas W. Kuyper

**Affiliations:** 1 Forest Ecology and Forest Management Group, Wageningen University, P.O. Box 47, 6700 AA Wageningen, The Netherlands; 2 Mekelle University, P.O. Box 231, Mekelle, Ethiopia; 3 Department of Soil Quality, Wageningen University, P.O. Box 47, 6700 AA Wageningen, The Netherlands; 4 Addis Ababa University, P.O. Box 1176, Addis Ababa, Ethiopia

**Keywords:** Water pulse, AM fungi, *Boswellia papyrifera*, Precipitation, Seedling

## Abstract

Under drought conditions, arbuscular mycorrhizal (AM) fungi alter water relationships of plants and improve their resistance to drought. In a factorial greenhouse experiment, we tested the effects of the AM symbiosis and precipitation regime on the performance (growth, gas exchange, nutrient status and mycorrhizal responsiveness) of *Boswellia papyrifera* seedlings. A continuous precipitation regime was imitated by continuous watering of plants to field capacity every other day during 4 months, and irregular precipitation by pulsed watering of plants where watering was switched every 15 days during these 4 months, with 15 days of watering followed by 15 days without watering. There were significantly higher levels of AM colonization under irregular precipitation regime than under continuous precipitation. Mycorrhizal seedlings had higher biomass than control seedlings. Stomatal conductance and phosphorus mass fraction in shoot and root were also significantly higher for mycorrhizal seedlings. Mycorrhizal seedlings under irregular watering had the highest biomass. Both a larger leaf area and higher assimilation rates contributed to higher biomass. Under irregular watering, the water use efficiency increased in non-mycorrhizal seedlings through a reduction in transpiration, while in mycorrhizal seedlings irregular watering increased transpiration. Because assimilation rates increased even more, mycorrhizal seedlings achieved an even higher water use efficiency. *Boswellia* seedlings allocated almost all carbon to the storage root. *Boswellia* seedlings had higher mass fractions of N, P, and K in roots than in shoots. Irregular precipitation conditions apparently benefit *Boswellia* seedlings when they are mycorrhizal.

**Electronic supplementary material:**

The online version of this article (doi:10.1007/s00442-012-2258-3) contains supplementary material, which is available to authorized users.

## Introduction

Prolonged seasonal drought severely affects regeneration of trees and survival of their seedlings and saplings in drylands (Gindaba et al. 2005; Gebrehiwot et al. 2005; Gebrekirstos et al. 2006). Arbuscular mycorrhizal (AM) fungi can alter water relationships of plants such that they improve the resistance of plants to drought (Augé 2001; Smith and Read 2008; Lambers et al. 2008; Ruiz-Lozano and Aroca 2010; Apple 2010). Various mechanisms have been proposed through which the AM symbiosis improves drought resistance. These underlying mechanisms are partly nutritional (enhanced uptake of P, but also of K, N, Ca, Mg, Zn, and Cu) and partly non-nutritional. Non-nutritional mechanisms include hormonal effects (through abscisic acid) due to mycorrhizal colonization, improved soil–hyphal contact (especially important during soil drying), more effective scavenging for water in micropores, direct water uptake by hyphae, and increased photosynthesis through sink stimulation (Augé 2001; Kaschuk et al. 2009; Smith et al. 2010). Augé (2004) subsequently demonstrated that the mycorrhizal network also affects moisture retention properties of soils, such that non-mycorrhizal plants growing in a mycorrhizal soil also had increased stomatal conductance. As Augé (2001) noted, field effects of AM fungi on increased plant performance under drought are usually a combination of nutritional and non-nutritional effects. While mycorrhizal plants thus acquire more water and nutrients, they inevitably transpire more water, too. However, several studies have also reported a mycorrhiza-induced increase in plant water use efficiency (WUE) (Kaya et al. 2003; Ruiz-Lozano and Aroca 2010).

In this study, we focused on the role of the AM symbiosis in the water relationships of the frankincense tree *Boswellia papyrifera* (Burseraceae—henceforth *Boswellia*). *Boswellia* is found in *Acacia*–*Commiphora* woodlands and wooded grasslands of dry areas in the Horn of Africa at 500–1,500 m altitude, with average temperatures of 20–25°C, annual rainfall below 900 mm, and with a wet growing season of 2–4 months (Bekele 2007; Ogbazgi et al. 2006). In these dry environments, high temperatures and erratic moisture inputs impose a pulsed pattern of water and soil nutrient availability between the dry season and wet growing season, but also between wetter and drier periods during the growth season (Collins et al. 2008; Schwinning and Sala 2004; Chesson et al. 2004). During the long dry season, which usually lasts for 8–10 months, seedlings naturally die-back above-ground but survive below-ground (Gindaba et al. 2004). Seedlings produce new above-ground shoots during the wet growing season, but may then still face water stress because of irregular rains. The precipitation regime thus involves both a long dry period and short term periods of water stress, and may select for specific plant morphological and physiological adaptations (Schwinning and Ehleringer 2001; Schwinning and Sala 2004). *Boswellia* is ecologically adapted to such pulsed growth conditions (Gebrehiwot et al. 2005; Ogbazgi et al. 2006; Abiyu et al. 2010). In an earlier study, we concluded that the AM symbiosis contributes to this adaptation and hence makes a major contribution to establishment, growth, and survival of this woodland species under the prevailing harsh climate (Birhane et al. 2010). Root colonization levels were higher during the dry than during the wet season. As the plants were leafless during the dry period (although *Boswellia* possesses photosynthetic stems), it seems plausible that carbon gain by the plant and carbon expenditure by the fungus are temporally disconnected. This temporal disconnect could be a specific adaptation to pulsed resource availability (Birhane et al. 2010). A temporal disconnect as a strategy of drought tolerance has been described before in the vernal herb *Erythronium americanum* (Lapointe and Molard 1997; Lapointe 2001). Querejeta et al. (2007b) described how hydraulic lift by woody roots of mycorrhizal trees resulted in water exudation from roots that allowed mycorrhizal fungal hyphae to remain active during periods of severe soil drying. In this study, we tested the effect of the AM symbiosis in combination with water availability (pulsed or not) for three *Boswellia* seedling age groups to determine seedling performance (carbon and nutrient acquisition, and water use). We hypothesized that:

- Mycorrhizal plants are larger than non-mycorrhizal plants;
- Mycorrhizal *Boswellia* seedlings have higher gas exchange, leaf water potential, and relative water content than seedlings without AM;
- Mycorrhizal benefit is larger under water-pulsed conditions (irregular water supply) than under conditions of regular watering;
- Higher biomass in mycorrhizal seedlings under water-pulsed conditions is a result of higher assimilation rate and water use efficiency.

## Materials and methods

A greenhouse experiment with *Boswellia* seedlings was conducted in northern Ethiopia at the Illala plant tissue culture greenhouse, Tigray Agricultural Research Institute, Mekelle (13°29′N, 39°28′E; altitude 2,000 m a.s.l.) from 1 June 2008 to 30 October 2009. Mean daily temperature of the greenhouse was 25°C during the day and 22°C during the night with mean daily average relative humidity of 51% for the study period.

### Seedling preparation and selection

Seeds from adult *Boswellia* trees from the dry deciduous woodlands in Abergelle, northern Ethiopia, were collected in March 2007. Healthy trees with a single stem and with uniform seed setting were selected for seed collection. Seeds were directly picked by hand from tree branches either by climbing or standing on the ground depending on tree height. Seeds were soaked in cold water for 12 h to accelerate germination. Germination took place in plastic trays filled with autoclaved pure river sand under greenhouse conditions. All seeds germinated within 5–10 days. A total of 160 germinated seeds were individually transplanted to plastic pots, 8 cm diameter and 15 cm high. Potted seedlings were placed on metal mesh benches and were watered regularly using micro-sprinkler irrigation every other day to field capacity until the plants were ready for the experiment. Dimethoate was sprayed to ward off ants and aphids which were observed on leaves. Of the 144 seedlings of uniform size, 12 seedlings were harvested after 1 month. At the same time, half of the remaining 132 seedlings were inoculated and all 132 were transplanted to larger perforated 20-l plastic containers. A single seedling was planted per container, each container being filled with 15 kg autoclaved soil.

### Preparation of inoculum of AM fungi

Spores of AM fungi were collected during the dry season from the rhizosphere of the same *Boswellia* trees by the wet sieving and decanting method (Brundrett et al. 1996). Most spores belonged to the genus *Glomus* (Birhane et al. 2010) and these were not further identified to species. Spore cultures were maintained on plants of *Sorghum bicolor*. The fungal inoculum added to the seedlings consisted of a mixture of soil, spores, and root fragments, produced from the rhizosphere of pre-colonized *Sorghum bicolor* plants. About 50 g of fungal inoculum was added near the roots of each seedling at the center of the pot. In order to mimic the natural growth conditions for the seedlings, the potting soil was also excavated from Abergelle, in a similar habitat where *Boswellia* trees naturally grow. Before inoculation, the potting soils were sieved and sterilized by an autoclave at 121°C for 2 h. Control seedlings were planted in sterilized soils.

### Experimental design and treatments

The experiment consisted of a three-factorial design: AM fungi (present or absent), water supply (continuous watering vs. pulsed watering), and seedling age (seedlings harvested after 4, 12, and 16 months). We mimicked seasonality by supplying water for 4 months followed by 8 months without any water. During that period, seedlings died back above-ground. After 1 year, we mimicked the second rainy season by supplying water for another 4 months. Seedlings harvested after 4 months were given water to field capacity; seedlings grown for 12 months were given water for 4 months, kept without water for 8 months, and harvested after re-emergence; and seedlings grown for 16 months were given water during the two rainy seasons, 4 months each, and left without water for 8 months. In order to simulate dry spells during the rainy season, we supplied the seedlings either with water for 15 days followed by 15 days of drought (water pulse, SP) or every other day (continuous water, WC). When watered, pots were filled to field capacity, based on their mass. The treatment units were arranged on greenhouse benches in a completely randomized design. There were 11 replications which gave a total of 144 seedlings (since 12 seedlings were initially harvested, 132 seedlings were actually planted and exposed to the actual treatments).

### Measurement of seedling traits

We harvested seedlings four times (at the start of the experiment, and after 4, 12, and 16 months). We determined plant size, biomass, and growth rate. Total shoot length (plant height) was measured using a graduated meter, and root collar diameter was measured using a digital caliper. The number of fully developed leaves was assessed for each seedling. Leaf surface area was measured using AM 100 leaf area meter (ADC Bioscientific). Harvested seedlings were divided into coarse roots, fine roots, stems, and leaves and their dry mass was determined after oven-drying at 80°C until constant weight was achieved. We then calculated leaf, stem, fine root, and coarse root mass fractions (dry mass·dry plant mass^−1^, g g^−1^], leaf area ratio [leaf area (cm^2^)·plant dry mass^−1^ (g^−1^)], specific leaf area [leaf area (cm^2^)·leaf dry mass^−1^ (g^−1^)], and root:shoot ratio (Hunt 1990). Total root length was estimated using the grid line intersect method (Tennant 1975). In addition, specific root length (root length·dry root mass^−1^, mm g^−1^), root length per unit plant mass and root length per unit leaf area were calculated as root traits. The number and length of primary roots per plant were assessed and determined. Relative growth rate was calculated according to Hunt (1990) and Chiariello et al. (1989).

### Plant nutrient analysis

Mineral status of the plants was determined by conducting shoot and root tissue elemental analysis. After sun-drying, shoot and root samples were oven-dried at 80°C for 48 h. Samples were then wet-digested and analyzed for N, P, and K. Total N was determined using the standard Kjeldahl method, P colorimetrically by spectrophotometer, and K by flame photometry (Anderson and Ingram 1993).

### Mycorrhizal colonization

Mycorrhizal colonization was assessed using the grid line intersection method (Giovannetti and Mosse 1980). Subsamples of (non-suberized) roots were collected, cleared with 10% KOH, and stained with 0.01% trypan blue in lactoglycerol (Brundrett et al. 1996). Roots were then divided into 1 cm pieces and mounted lengthwise on a microscope slide. Eleven slides per treatment per harvest containing nine root pieces per slide were examined by making three microscope observations (top, middle and bottom) per 1 cm root piece at ×400 magnification (*n* = 891). Colonization was expressed as percentage of the root length colonized. Total fractional colonization and those of arbuscules, vesicles, and internal hyphae in the root cortex were recorded.

Mycorrhizal responsiveness was expressed as the ratio of total dry weight of mycorrhizal plants and non-mycorrhizal plants. Drought response index was calculated as the ratio of total dry weight of plants exposed to water supplied in pulses (SP) to plants under well-watered conditions (WC).

### Gas exchange measurements

Gas exchange was measured for the 16-month-old seedlings. Measurements were made between 0900 and 1200 hours from five mature fully expanded leaves two times per leaf (*n* = 11) under full sunlight using a LCP-002 portable photosynthesis system (LC Pro; ADC Bioscientific). Measurements included net photosynthesis rate, stomatal conductance, transpiration rate, and dark respiration rate. Photosynthetic water use efficiency was calculated as the ratio between the photosynthesis rate and transpiration rate. Predawn (0300–0600 hours) and midday (1200–1400 hours) leaf water pressure potential were measured using a pressure chamber apparatus (Scholander et al. 1965) using well-expanded leaves. Relative water content of leaves was measured according to Koide et al. (1989). Water pressure potential and relative water content were determined twice in the course of the experiment.

### Statistical analysis

A three-way analysis of variance (ANOVA) was used to test for significant sources of variation in differences in seedling size, biomass and nutrient levels; a two-way ANOVA was applied for root colonization (water pulse; seedling age) and gas exchange (water pulse, mycorrhiza). Repeated-measures ANOVA was used to analyze treatment effects on predawn and midday relative water content and leaf water potential of seedlings. Because of the large number of parameters, a sequential Bonferroni correction was applied at *P* < α/(1 + *k* − *i*) significance level, with α = 0.05; *k* is the number of parameters (40); *i* is the sequential value after sorting in ascending order, when *F* test from ANOVA was significant. Gabriel post hoc test for unequal sample size and LSD for main effect comparison were performed. In order to meet the assumptions of normal distribution and homogeneity of variances, data on leaf number were arcsine square root-transformed; data on root collar diameter, shoot length, stem dry mass, coarse root dry mass, fine root dry mass, shoot dry mass, total plant dry mass, leaf relative growth rate, root length per leaf area, net photosynthetic rate, and dark respiration rate were log-transformed before statistical analysis. Treatment effects were statistically analyzed using SPSS (PASW statistics 17) software.

## Results

### Plant size and growth

**Fig. 1:**
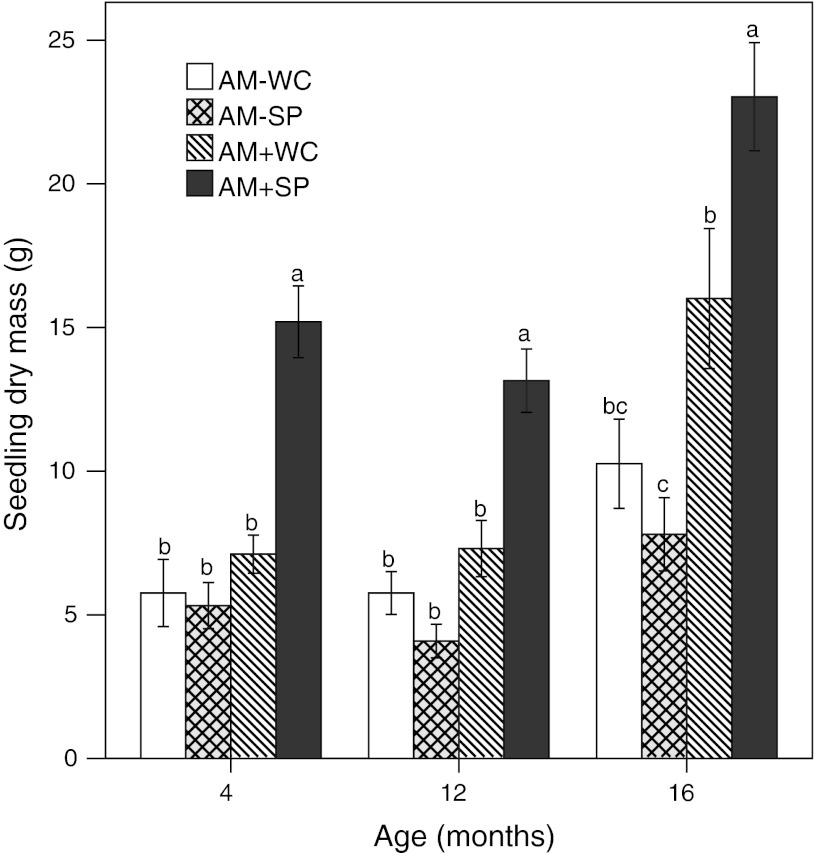
The effects is presented of arbuscular mycorrhiza and water regime on seedling mass (mean ± 1SE) of *Boswellia*. Arbuscular mycorrhizal treatment (AM+) is compared with a control without inoculation (AM−), and water pulse every 2 weeks (SP) during the wet seasons is compared with a control with regular water supply during the wet season (WC). Seedlings in the 12-month category die back aboveground during the 8-month dry period, as a consequence of which there were no differences in biomass between the 4- and 12-month-old seedlings. *Different letters* (per age class) indicate significant differences between treatments (*P* < 0.05)

**Fig. 2:**
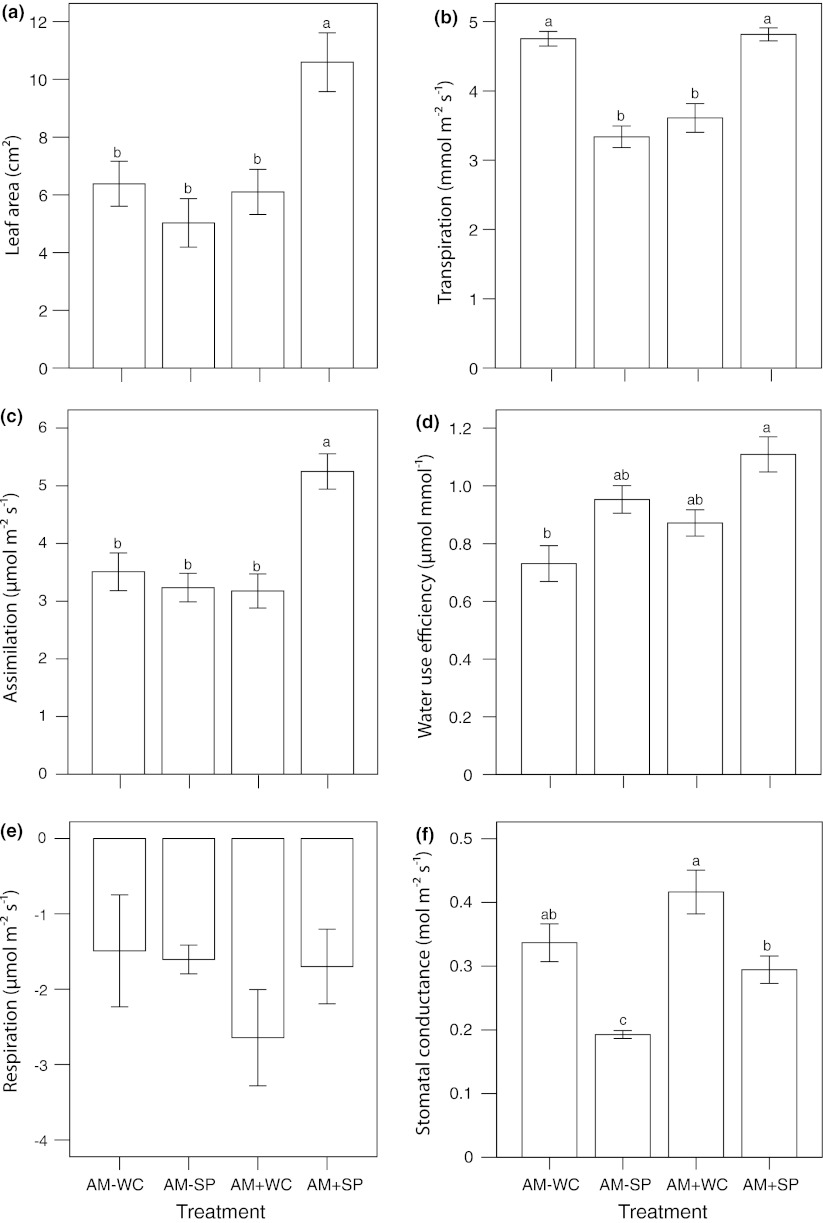
The effects is presented of arbuscular mycorrhiza and water regime on gas exchange (mean ± 1SE) of 16-month-old *Boswellia* seedlings. Arbuscular mycorrhizal treatment (AM+) is compared with a control without inoculation (AM−), and water pulse every 2 weeks (SP) during the wet seasons is compared with a control with regular water supply during the wet season (WC). *Different letters* indicate significant differences between treatments (*P* < 0.05)

Most plant traits were significantly affected by seedling age, and some (especially root biomass) by mycorrhiza, while water pulse was in most cases not a significant source of variation. The interaction water pulse × mycorrhiza was also significant for several plant traits, again those related to below-ground performance (Table 1). *Boswellia* seedlings were mycorrhiza-responsive. Because drought response index was smaller than 1 in non-mycorrhizal seedlings but larger than 1 in mycorrhizal seedlings, mycorrhizal responsiveness was much higher after water pulsing than with continuous water supply (Fig. 1). For the three age classes, mycorrhizal responsiveness was around 300% for seedlings exposed to water-pulsing, whereas it was around 150% for seedlings with continuous water supply. Mycorrhizal responsiveness increased with the duration of the experiment. Both a larger leaf area (Table 1; Fig. 2a; significant interaction water pulse × mycorrhiza) and higher assimilation rate per unit leaf area (Table 2; Fig. 2c; water pulse, mycorrhiza and interaction water pulse × mycorrhiza all significant sources of variation) contributed to this increased plant dry mass. Higher assimilation rate per unit leaf area coincided with higher P mass fractions in mycorrhizal seedlings.

**Table 1:** ANOVA table showing the effect of mycorrhiza and water pulse on plant traits of *Boswellia* seedlings

Parameters	Units	AM		Water		AM × water	
		*F*	*P*	*F*	*P*	*F*	*P*
Leaf number	Number	36.341	0.000*	0.105	0.747	0.006	0.940
Leaf area	cm^2^	3.891	0.052	1.977	0.163	9.817	0.001*
Coarse root dry mass	g	110.464	0.000*	9.342	0.003	31.221	0.000*
Root dry mass	g	107.133	0.000*	9.404	0.003	31.084	0.000*
Plant dry mass	g	98.902	0.000*	7.599	0.007	30.021	0.000*
Biomass increase	%	45.520	0.000*	0.384	0.537	8.421	0.004
Plant relative growth rate	% month^−1^	94.144	0.000*	6.200	0.014	27.247	0.000*
Root relative growth rate	% month^−1^	101.055	0.000*	7.624	0.007	27.514	0.000*
Coarse root relative growth rate	% month^−1^	104.123	0.000*	7.532	0.007	27.583	0.000*
Specific root length	mm g^−1^	17.994	0.000*	1.531	0.218	0.473	0.493
Root length per plant mass	mm g^−1^	17.113	0.000*	0.730	0.395	0.751	0.388
Shoot phosphorus	%	13.527	0.000*	9.614	0.003	1.973	0.163
Root phosphorus	%	13.888	0.000*	7.697	0.007	0.019	0.889

A two-way ANOVA was used to test the effect of mycorrhiza, watering and their interaction but only presented for parameters with significant effect (the whole effect was presented as an electronic supplementary table). Except for gas exchange, all traits were measured and/or calculated after the harvest

* Significant after Bonferroni correction

**Table 2:** Fractional mycorrhizal colonization and mycorrhizal root length (mean ± SE) for inoculated seedlings of *Boswellia*

Colonization percentage	Age					Water				Age × water	
	4	12	16	*F*	*P*	WC	SP	*F*	*P*	*F*	*P*
Arbuscular colonization	0.404 ± 0.03	0.464 ± 0.03	0.577 ± 0.03	1.953	0.151	0.344 ± 0.02b	0.62 ± 0.02a	14.815	0.000	0.315	0.731
Vesicular colonization	0.631 ± 0.03	0.637 ± 0.03	0.657 ± 0.03	0.054	0.948	0.448 ± 0.02b	0.814 ± 0.02a	29.148	0.000	4.795	0.012
Hyphal colonization	0.649 ± 0.04b	0.725 ± 0.04b	0.915 ± 0.04a	7.443	0.001	0.641 ± 0.03b	0.885 ± 0.03a	15.596	0.000	0.094	0.911
Mycorrhizal root length	13.091 ± 1.333	21.281 ± 1.333	32.35 ± 1.333	2.470	0.093	13.489 ± 1.264b	32.137 ± 1.333a	6.790	0.012	0.957	0.390

A two-way ANOVA is used to test for the effects of age and water and their interaction

Different letters indicate significant differences between treatments (*P* < 0.05)

*Boswellia* seedlings allocated most carbon below-ground, especially to the coarse root, regardless of treatment, and age. Coarse-root mass fraction was 83% for non-mycorrhizal seedlings and somewhat higher, 92% for mycorrhizal seedlings. The interaction water pulse × mycorrhiza was significant.

### Plant nutrients

Nutrient mass fractions in shoot and root were significantly affected by seedling age (for N, P, K), mycorrhiza (for P), but not by water pulse (online information 1). Phosphorus mass fractions in shoot and roots were significantly higher for AM seedlings. The interaction mycorrhiza × age was significant for shoot N, and shoot and root P while the interaction age × water pulse was significant for root N and K (online information 1). *Boswellia* seedlings had significantly higher mass fractions of N and K in their roots than in the shoots (*P* < 0.001).

### AM colonization

All control *Boswellia* seedlings at all harvests remained free of mycorrhiza. Water pulse was a significant source of variation for fractional colonization (Table 2). Average fractional colonization for the seedlings under continuous water supply was 64%, compared to 89% for the seedlings that received a water pulse. Arbuscular, vesicular, and hyphal colonization and mycorrhizal root length were all significantly higher for seedlings in the water-pulse treatment than in the regular water treatment (Table 2).

### Gas exchange

Seedling age, mycorrhiza, and water pulse were all significant sources of variation for stomatal conductance, whereas none of the interactions were significant (online resource 1). Assimilation and water use efficiency of the oldest seedlings were significantly affected by mycorrhiza, water pulse, and the interaction (Table 3). Transpiration rate was not affected by mycorrhiza or water pulse, but the interaction was highly significant. Mycorrhizal plants in the water-pulse treatment had significantly higher assimilation rates than the other treatments (Fig. 2c). Transpiration rates were significantly higher in non-mycorrhizal plants with regular water supply and in mycorrhizal plants under pulsed conditions than in the other treatments (Fig. 2b). Water use efficiency was higher for mycorrhizal than for non-mycorrhizal plants and higher for water-pulsed plants than for plants that received water regularly (Fig. 2d). Stomatal conductance was higher for mycorrhizal plants than for non-mycorrhizal plants and higher for regularly watered plants than for plants in the water-pulse treatment (Fig. 2f). Stomatal conductance and water use efficiency were not correlated. No significant differences were observed in relative water content and leaf water potential among treatments within the same age class (online resource 1).

**Table 3:** Gas exchange (mean ± SE) in 16-month-old *Boswellia* seedlings

Traits	Units	AM inoculation				Water				AM × water	
		AM−	AM+	*F*	*P*	WC	SP	*F*	*P*	*F*	*P*
Assimilation	μmol C m^−2^ s^−1^	2.88 ± 1.057b	3.72 ± 1.069a	8.466	0.004	2.95 ± 1.073	3.66 ± 1.049	5.539	0.020	8.116	0.005
Stomatal conductance	mol m^−2^ s^−1^	0.254 ± 0.017b	0.344 ± 0.018a	13.777	0.000	0.361 ± 0.018a	0.237 ± 0.016b	25.929	0.000	0.684	0.409
Transpiration	mmol H_2_O m^−2^ s^−1^	4.046 ± 0.093	4.214 ± 0.111	1.345	0.248	4.182 ± 0.120	4.077 ± 0.081	0.519	0.472	82.019	0.000
Respiration	μmol C m^−2^ s^−1^	−0.73 ± 1.230	−1.285 ± 1.294	2.837	0.097	−0.75 ± 1.23	−1.26 ± 1.291	2.490	0.120	4.074	0.048
Water use efficiency	μmol mmol^−1^	0.842 ± 0.044b	0.990 ± 0.053a	4.663	0.032	0.801 ± 0.057b	1.031 ± 0.039a	11.193	0.001	7.380	0.000

A two-way ANOVA is used to test for the effects of mycorrhiza and water and their interaction

Different letters indicate significant differences between treatments (*P* < 0.05)

## Discussion

We observed a positive effect of the mycorrhizal symbiosis on the growth and biomass of *Boswellia* seedlings. The high mycorrhizal responsiveness of *Boswellia,* especially under conditions of water-pulsing, confirms the crucial role of the mycorrhizal symbiosis in these harsh environments. The positive mycorrhizal effect is due to improved phosphorus nutrition, which was evident by the significantly higher P mass fractions in shoots and roots of mycorrhizal plants compared to non-mycorrhizal plants. The beneficial effect of the mycorrhizal symbiosis was much larger under irregular precipitation (or water-pulsed) conditions than regular precipitation (continuous water supply).

Irregular water supply had a negative effect in non-mycorrhizal *Boswellia* seedlings. However, water-pulsing improved performance of mycorrhizal seedlings compared to regular watering, and this effect became stronger over time. This contrast confirms the essential role of the mycorrhizal symbiosis in case of unpredictable water availability. Both leaf area and assimilation rate per unit leaf area were highest in that treatment combination. The AM symbiosis increased coarse root biomass resulting in more water storage (Roumet et al. 2006; Chidumayo and Frost 1996). Plants that store more water can maintain their stomata open, resulting in increased assimilation and (but to a lesser extent) increased transpiration rates (Fig. 2b, c). Mycorrhizal fungi in combination with water supply in pulses drove some of the major changes in leaf area, coarse root mass, and leaf photosynthesis in *Boswellia* seedlings, which positively influence growth (Daughtridge et al. 1986; Wright et al. 1998).

In conjunction with larger plant size and an improved phosphorus status of mycorrhizal plants, we noted an increase in assimilation (in combination with water-pulsing), water use efficiency, and stomatal conductance (both independent of water pulsing; Fig. 2). An increase in stomatal conductance in mycorrhizal plants has been repeatedly observed. Such increases were noted both for comparisons of mycorrhizal and non-mycorrhizal plants of the same size and P status and for cases where mycorrhizal plants were significantly larger and with higher leaf nutrient mass fractions (Augé 2001). Subsequent studies have confirmed this pattern, while also showing the importance of plant functional type. Apparently, plant strategy (along the axis from a conservative to acquisitive strategy for both above-ground and below-ground resources; cf. Díaz et al. 2004) is a major determinant of the extent to which the mycorrhizal symbiosis affects stomatal conductance. Querejeta et al. (2003) observed that AM fungi enhanced stomatal conductance in the slow-growing *Olea europaea* ssp. *sylvestris* and hardly so in the fast-growing *Rhamnus lycioides*. In a subsequent study, Querejeta et al. (2007a) compared seedlings of the dryland shrubs *Pistacia lentiscus* and *Retama sphaerocarpa*, a leafless legume with photosynthesizing stems. *Retama* was less responsive to mycorrhizal inoculation than *Pistacia* and showed no increase in stomatal conductance (as assessed from the δ^18^O signal, a proxy for stomatal conductance). However, the mycorrhizal symbiosis can also lower stomatal conductance. Goicoechea et al. (2004) reported that drought induced a larger decline in stomatal conductance in the drought-avoiding, nitrogen-fixing *Anthyllis cytisoides* in the mycorrhizal condition than in the non-mycorrhizal condition. Drought also reduced photosynthesis more in mycorrhizal than in non-mycorrhizal plants, and leaf nutrient mass fractions were also lower in mycorrhizal plants at peak drought. The authors concluded that the mycorrhizal symbiosis still conferred drought resistance to this shrub, because mycorrhizal plants had higher rates of leaf shedding as a mechanism of adaptation to drought. Not only plant identity but also fungal identity could affect changes in stomatal conductance under drought. Querejeta et al. (2006) compared native, drought-adapted AM fungi with non-native strains and noted that the drought-adapted species more strongly increased stomatal conductance than non-native species. Larger increases in stomatal conductance in drought-adapted AM fungal species coincided with larger improvements in nutrient uptake. A major effect of AM fungal species provenance was also noted by Ruiz-Lozano and Azcon (1995) and Marulanda et al. (2006).

Increased stomatal conductance of mycorrhizal plants compared to non-mycorrhizal plants would normally translate into increased photosynthesis. However, this response was not observed when plants were adequately watered (WC). Instead, transpiration rates of mycorrhizal plants were significantly lower than those of non-mycorrhizal plants, and as a consequence the mycorrhizal symbiosis did improve water use efficiency. Under water-pulsed conditions (SP), increased stomatal conductance resulted in increased assimilation rates. Transpiration rates also increased, but the fractional increase was smaller. Consequently, water use efficiency was also higher for mycorrhizal plants compared to non-mycorrhizal plants under conditions of pulsed water availability.

Even though *Boswellia* seedlings always allocated most carbon to the coarse root system, mycorrhizal seedlings had more C allocated to the roots than non-mycorrhizal seedlings. The significantly higher amounts of carbon and nutrients allocated below-ground indicate that AM *Boswellia* seedlings store the resources acquired during the growing season in the roots when they die back as a resource conservation strategy. Considering the structure of the coarse root, it is likely that this organ also serves as a water storage organ from which mycorrhizal fungi might benefit under conditions of drought. Querejeta et al. (2007b) studied the effect of severe soil drying on the functioning of the mycorrhizal fungal network in an oak savanna in California. The authors concluded that the trees access water from groundwater, and that this water is exuded in the topsoil from which the mycorrhizal fungal mycelium subsequently benefits. In our greenhouse, the seedlings did not have access to additional water sources. However, storage of water in coarse roots and its provision to the AM fungi explain why mycorrhizal plants did have access to more water during an irregular precipitation regime. Moreover, mycorrhizal plants under water-pulsed conditions had significantly higher transpiration rates than those of plants provided with regular water. Provision of water to hyphae through the coarse roots also explains how mature trees maintain (and even increase) levels of mycorrhizal colonization during dry periods, as observed in our previous field study (Birhane et al. 2010).

A better water status of mycorrhizal seedlings compared to non-mycorrhizal seedlings may result in seedlings that are better able to capture resources during the next rainy season. After a period of 8 months of not watering, when seedlings had died back above-ground, an increase in atmospheric humidity resulted in renewed above-ground emergence of seedlings. Similar behavior to increased atmospheric humidity was observed in miombo trees (Chidumayo and Frost 1996). Seedling emergence, before the next round of watering started, was higher for mycorrhizal seedlings (53%) compared to non-mycorrhizal seedlings (37%), but the difference was not significant. Faster emergence expands the window of opportunity for *Boswellia* seedlings to further achieve a positive carbon and nutrient balance, and may ultimately shorten the period (of several years to decades) that the tree shows annual cycles of die-back, before it finally achieves the height growth that allows it to escape from grazing and fire.

## Conclusion

The AM symbiosis changed plant growth, biomass (especially below-ground biomass), phosphorus mass fraction in leaves and roots, and photosynthetic performance of *Boswellia* seedlings. The strong interaction between mycorrhiza and the water-pulse treatment showed that mycorrhizal *Boswellia* actually benefits from irregular water supply during the short rainy season. Water-pulsing increased leaf area and phosphorus mass fractions in mycorrhizal seedlings, resulting in the highest assimilation rates. Even though transpiration rates were also increased, water use efficiency was highest in mycorrhizal seedlings in the irregular precipitation regime. The conservative acquisition strategy of *Boswellia*, where carbon and water acquired in the rainy season are stored in coarse roots, is beneficial in this harsh climate. By this strategy (waiting in the underground), seedlings disconnect carbon gain from nutrient gain, and store reserves below-ground until they are able to produce shoots that grow sufficiently high to escape the impacts of grazing and fire. A major question for further research is whether other trees than *Boswellia* in this habitat show this conservative acquisition strategy, or whether more acquisitive strategies also occur under such climates. A further question is how mycorrhiza and water availability affect competition between plants with different resource acquisition strategies. Such knowledge is essential for sustainable management of these economically and ecologically important species.

## Electronic supplementary material

Below is the link to the electronic supplementary material.

Supplementary material 1 (DOCX 28 kb)
