# Supplementary material for: Arbuscular mycorrhizal fungi enhance photosynthesis, water use efficiency, and growth of frankincense seedlings under pulsed water availability conditions
Source: Oecologia. 2012 Jan 28;169(4):895–904. doi: 10.1007/s00442-012-2258-3 (PMC3398253; doi:10.1007/s00442-012-2258-3)
Supplement: Supplementary file 1 — Supplementary material 1 (DOCX 28 kb) [file 442_2012_2258_MOESM1_ESM.docx]

Arbuscular mycorrhizal fungi enhance the photosynthesis water use efficiency and growth of frankincense seedlings under pulsed water availability conditions

Emiru Birhane^a,b,c^, Frank J. Sterck^a^ , Masresha Fetene^d^, Frans Bongers^a^, Thomas W. Kuyper^c^

^a^ Wageningen University, Forest Ecology and Forest Management Group, P.O. Box 47, 6700 AA, Wageningen, The Netherlands

^b^ Mekelle University, P.O. Box 231, Mekelle, Ethiopia

^c^ Wageningen University, Department of Soil Quality, P.O. Box 47, 6700 AA Wageningen, The Netherlands

^d^ Addis Ababa University, P.O. Box, 1176, Addis Ababa, Ethiopia

Corresponding author

e-mail: [Frank.Sterck@wur.nl](mailto:Frank.Sterck@wur.nl)

telephone: +317-487399

fax: Fax +31 317 47878

ESM 1. ANOVA table showing the effect of age, mycorrhiza, and water pulse on plant traits of *Boswellia* seedlings. Except for gas exchange, all traits were measured and/or calculated after the harvest. A three-way ANOVA was used to test the effect of age, mycorrhiza and water

| **Parameters** | **Units** | **Age** | | **AM** | | **Water** | | **Age x AM** | | **Age x water** | | **AM x water** | | **Age x AM x water** | |
| --- | --- | --- | --- | --- | --- | --- | --- | --- | --- | --- | --- | --- | --- | --- | --- |
|  |  | F | P | F | p | F | p | F | p | F | p | F | p | F | p |
| Root collar diameter | mm | 4.336 | 0.016 | 6.354 | 0.013 | 0.372 | 0.544 | 0.449 | 0.640 | 1.933 | 0.150 | 2.357 | 0.128 | 1.698 | 0.189 |
| Height | mm | 43.221 | 0.000* | 8.861 | 0.004 | 0.174 | 0.678 | 1.339 | 0.267 | 1.157 | 0.319 | 0.567 | 0.453 | 0.530 | 0.590 |
| Leaf number | number | 260.006 | 0.000* | 36.341 | 0.000* | 0.105 | 0.747 | 10.297 | 0.000* | 0.254 | 0.776 | 0.006 | 0.940 | 0.426 | 0.654 |
| Leaf area | cm^2^ | 18.553 | 0.000* | 3.891 | 0.052 | 1.977 | 0.163 | 3.595 | 0.032 | 1.476 | 0.234 | 9.817 | 0.001* | 1.694 | 0.190 |
| Coarse root length | mm | 70.279 | 0.000* | 0.619 | 0.433 | 0.107 | 0.744 | 0.367 | 0.693 | 0.501 | 0.607 | 0.423 | 0.517 | 0.296 | 0.744 |
| Coarse root diameter | mm | 45.546 | 0.000* | 0.057 | 0.812 | 0.006 | 0.937 | 0.227 | 0.797 | 0.205 | 0.815 | 0.652 | 0.421 | 0.057 | 0.944 |
| Fine root length | mm | 7.487 | 0.001* | 0.004 | 0.952 | 1.258 | 0.264 | 0.876 | 0.419 | 0.887 | 0.414 | 0.241 | 0.625 | 0.984 | 0.377 |
| Root branch number | number | 3.961 | 0.022 | 0.395 | 0.531 | 0.864 | 0.355 | 0.113 | 0.893 | 0.355 | 0.702 | 0.019 | 0.892 | 0.942 | 0.393 |
| Leaf dry mass | g | 11.014 | 0.000* | 2.066 | 0.154 | 0.730 | 0.395 | 5.206 | 0.007 | 0.853 | 0.430 | 0.009 | 0.926 | 0.698 | 0.500 |
| Stem dry mass | g | 18.873 | 0.000* | 0.605 | 0.439 | 0.000 | 0.989 | 4.562 | 0.013 | 3.025 | 0.053 | 5.714 | 0.019 | 0.611 | 0.545 |
| Coarse root dry mass | g | 23.355 | 0.000* | 110.464 | 0.000* | 9.342 | 0.003 | 0.105 | 0.901 | 1.148 | 0.321 | 31.221 | 0.000* | 0.000 | 1.000 |
| Fine root dry mass | g | 14.795 | 0.000* | 4.128 | 0.044 | 0.000 | 0.999 | 0.768 | 0.466 | 0.320 | 0.727 | 0.164 | 0.686 | 5.950 | 0.003 |
| Shoot dry mass | g | 12.585 | 0.000* | 8.309 | 0.005 | 0.095 | 0.759 | 3.426 | 0.036 | 3.687 | 0.028 | 5.085 | 0.026 | 0.391 | 0.677 |
| Root dry mass | g | 24.297 | 0.000* | 107.133 | 0.000* | 9.404 | 0.003 | 0.077 | 0.926 | 1.011 | 0.367 | 31.084 | 0.000* | 0.016 | 0.984 |
| Plant dry mass | g | 26.998 | 0.000* | 98.902 | 0.000* | 7.599 | 0.007 | 0.271 | 0.763 | 1.209 | 0.302 | 30.021 | 0.000* | 0.176 | 0.839 |
| Biomass increase | % | 11.270 | 0.000* | 45.520 | 0.000* | 0.384 | 0.537 | 1.912 | 0.152 | 0.596 | 0.553 | 8.421 | 0.004 | 0.657 | 0.520 |
| Plant relative growth rate | %.month^-1^ | 23.606 | 0.000* | 94.144 | 0.000* | 6.200 | 0.014 | 0.102 | 0.903 | 1.166 | 0.315 | 27.247 | 0.000* | 0.246 | 0.783 |
| Shoot relative growth rate | %.month^-1^ | 5.517 | 0.005* | 5.194 | 0.025 | 0.179 | 0.673 | 1.249 | 0.291 | 5.347 | 0.006 | 6.903 | 0.010 | 1.087 | 0.341 |
| root relative growth rate | %.month^-1^ | 20.571 | 0.000* | 101.055 | 0.000* | 7.624 | 0.007 | 0.069 | 0.933 | 0.936 | 0.395 | 27.514 | 0.000* | 0.030 | 0.971 |
| Leaf relative growth rate | %.month^-1^ | 1.854 | 0.165 | 0.066 | 0.798 | 0.466 | 0.497 | 1.585 | 0.213 | 1.410 | 0.252 | 0.001 | 0.979 | 0.059 | 0.943 |
| Stem relative growth rate | %.month^-1^ | 9.836 | 0.000* | 0.259 | 0.612 | 0.016 | 0.901 | 5.244 | 0.007 | 7.806 | 0.001* | 4.815 | 0.031 | 0.558 | 0.574 |
| Coarse root relative growth rate | %.month^-1^ | 18.933 | 0.000* | 104.123 | 0.000* | 7.532 | 0.007 | 0.066 | 0.937 | 1.078 | 0.343 | 27.583 | 0.000* | 0.004 | 0.996 |
| Fine root relative growth rate | %.month^-1^ | 12.221 | 0.000* | 9.805 | 0.002 | 0.007 | 0.934 | 0.102 | 0.904 | 0.027 | 0.973 | 1.907 | 0.170 | 8.960 | 0.000* |
| Leaf area ratio | cm^2^g^-1^ | 7.388 | 0.001* | 7.763 | 0.006 | 0.340 | 0.561 | 5.885 | 0.004 | 0.307 | 0.736 | 0.705 | 0.403 | 0.681 | 0.508 |
| Specific leaf area | cm^2^g^-1^ | 7.458 | 0.001* | 0.045 | 0.833 | 0.262 | 0.610 | 0.602 | 0.550 | 1.069 | 0.347 | 1.080 | 0.302 | 0.854 | 0.429 |
| Root to shoot ratio | gg^-1^ | 0.894 | 0.412 | 7.603 | 0.007 | 3.281 | 0.073 | 4.272 | 0.016 | 6.673 | 0.002 | 0.688 | 0.408 | 0.956 | 0.387 |
| Specific root length | mm g^-1^ | 10.437 | 0.000* | 17.994 | 0.000* | 1.531 | 0.218 | 5.958 | 0.003 | 1.326 | 0.269 | 0.473 | 0.493 | 1.611 | 0.204 |
| Root length per plant mass | mm g^-1^ | 10.567 | 0.000* | 17.113 | 0.000* | 0.730 | 0.395 | 5.803 | 0.004 | 0.499 | 0.609 | 0.751 | 0.388 | 0.639 | 0.530 |
| Root length per leaf area | mm cm^-2^ | 9.124 | 0.000* | 0.026 | 0.872 | 0.022 | 0.882 | 0.949 | 0.391 | 1.377 | 0.258 | 0.422 | 0.518 | 2.856 | 0.063 |
| Pre-down relative water content | % | 244.871 | 0.000* | 0.008 | 0.930 | 2.345 | 0.130 | 5.150 | 0.026 | 0.000 | 1.000 | 2.368 | 0.128 | 1.746 | 0.190 |
| Mid-day relative water content | % | 184.994 | 0.000* | 2.223 | 0.140 | 0.309 | 0.580 | 0.026 | 0.872 | 0.181 | 0.671 | 1.782 | 0.186 | 1.667 | 0.200 |
| Pre-down leaf water potential | bars | 23.202 | 0.000* | 1.535 | 0.219 | 0.155 | 0.694 | 3.343 | 0.071 | 5.807 | 0.018 | 1.187 | 0.279 | 0.897 | 0.346 |
| Mid-day leaf water potential | bars | 18.827 | 0.000* | 0.003 | 0.960 | 1.042 | 0.310 | 9.056 | 0.004 | 4.205 | 0.044 | 0.603 | 0.440 | 4.051 | 0.048 |
| Stomatal conductance | mmol m^-2^ s^-1^ | 15.072 | 0.000* | 13.777 | 0.000* | 25.929 | 0.000* | 0.276 | 0.600 | 9.964 | 0.002 | 0.684 | 0.409 | 5.018 | 0.026 |
| Shoot Nitrogen | % | 6.207 | 0.003* | 0.930 | 0.337 | 0.100 | 0.752 | 10.495 | 0.000* | 5.717 | 0.005 | 0.333 | 0.565 | 6.120 | 0.003 |
| Root Nitrogen | % | 45.861 | 0.000* | 5.862 | 0.017 | 4.329 | 0.040 | 0.529 | 0.591 | 10.509 | 0.000* | 0.320 | 0.573 | 2.367 | 0.099 |
| Shoot Phosphorus | % | 32.617 | 0.000* | 13.527 | 0.000* | 9.614 | 0.003 | 12.928 | 0.000* | 5.463 | 0.006 | 1.973 | 0.163 | 5.086 | 0.008 |
| Root Phosphorus | % | 64.023 | 0.000* | 13.888 | 0.000* | 7.697 | 0.007 | 9.703 | 0.000* | 1.847 | 0.163 | 0.019 | 0.889 | 0.242 | 0.785 |
| Shoot Potassium | % | 156.679 | 0.000* | 0.850 | 0.359 | 5.591 | 0.020 | 7.805 | 0.001* | 2.006 | 0.140 | 0.056 | 0.814 | 13.358 | 0.000* |
| Root Potassium | % | 262.489 | 0.000* | 2.425 | 0.122 | 8.366 | 0.005 | 0.810 | 0.448 | 8.105 | 0.001* | 0.220 | 0.640 | 0.517 | 0.598 |

*Significant after Bonferronni correction
